# Composite Resection of Tumors of the Rostral Maxilla and Dorsolateral Muzzle Utilizing an Upper Lip-Sparing, Combined Approach in Dogs

**DOI:** 10.3389/fvets.2018.00054

**Published:** 2018-03-20

**Authors:** Amy E. Thomson, Jason W. Soukup

**Affiliations:** ^1^Dentistry and Oral Surgery, Department of Surgical Sciences, School of Veterinary Medicine, University of Wisconsin-Madison, Madison, WI, United States

**Keywords:** oral tumors, dog, composite resection, combined approach, lip sparing, surgical oncology, fibrosarcoma

## Abstract

Tumors of the rostral maxilla that involve both the oral mucosa and the dermis or subdermis of the dorsolateral muzzle provide unique challenges for the oromaxillofacial surgeon. Traditionally described approaches to such lesions may involve an intraoral incision that extends and involves the upper lip to envelope the involved dermis of the dorsolateral muzzle. However, such an approach unnecessarily resects upper lip tissue resulting in a large defect that likely requires advanced skin flaps or grafts for reconstruction. Such flaps are technically challenging and introduce potential for significance postoperative complications. In this article, we provide a detailed description a combined intra- and extraoral approach that allows for composite resection of tumors of the rostral maxilla that also involve the dorsolateral muzzle. The described technique allows for excellent intraoperative visualization and provides a superior cosmetic outcome that minimizes postoperative complications. In addition, we describe our experience utilizing the technique in three clinical cases.

## Introduction

The goal of any tumor resection is to obtain tumor-free margins while maintaining proper function and, ideally, achieving an excellent cosmetic outcome. Achievement of these goals is dependent on tumor location, invasiveness, and the complexity of the resultant wound closure. Rostral maxillary tumors that also involve the nasal bone and overlying skin of the dorsolateral muzzle often require more complicated composite resections. Composite resections are defined as en bloc resections involving adjacent tissue types (e.g., skin and underlying bone). From dorsal to ventral, composite resections of the rostral and dorsolateral maxilla entail en bloc resection of skin, nasal bone, ±incisive bone, nasal mucosa and turbinates, maxilla bone, hard palate, teeth, gingiva, and oral mucosa. Because of the intra- and extraoral nature of tumors of the rostral maxilla and dorsolateral muzzle, the surgical approach is often more complicated than the more straightforward intraoral tumors that do not involve the dorsolateral muzzle. The surgical closure of the resultant wound, which often necessitates reconstruction of lip tissue, may also present additional challenges and surgical complications. In addition, postoperative function and cosmetic outcome may be less than desirable. In such situations, a combined intra- and extraoral approach to composite resections of tumors of the rostral maxilla and dorsolateral muzzle that spares the lip may be utilized. Such a technique preserves an isthmus of upper lip, which allows good to excellent cosmesis and decreased postoperative morbidity as no axial pattern flap is required for lip reconstruction. Although this technique has been very briefly described in a textbook, to the authors’ knowledge, a detailed description of the application of this technique in clinical patients has not previously been described in the peer-reviewed English language literature. Therefore, we report our experience utilizing the upper lip-sparing composite resection of rostral maxilla and dorsolateral muzzle tumors utilizing a combined extra- and intraoral approach and postoperative outcome in three dogs.

## Surgical Technique

**Figure 1:**
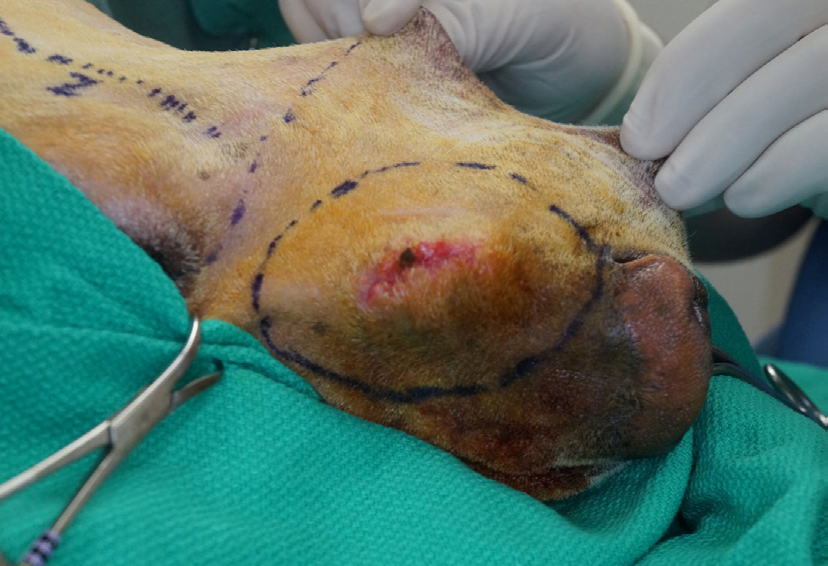
Intraoperative photograph of the dermal incision in case #1 depicting the extraoral approach that allows the composite resection of the lesion.

**Figure 2:**
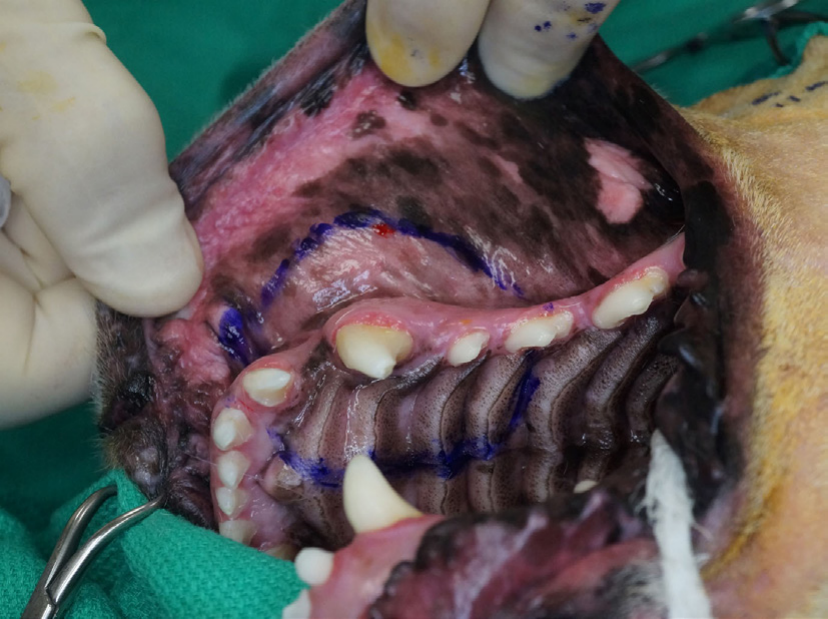
Intraoperative photograph of the mucosal incision in case #1 depicting the intraoral approach that allows the composite resection of the lesion.

**Figure 3:**
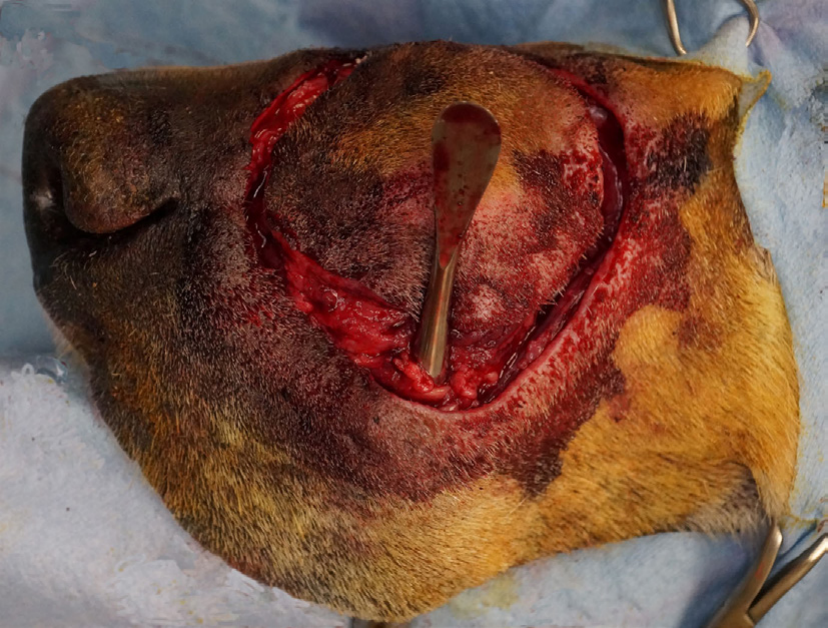
Intraoperative photograph revealing the communication of the extraoral and intraoral incisions. Note the periosteal elevator placed through the intraoral incision to exit the extraoral incision.

**Figure 4:**
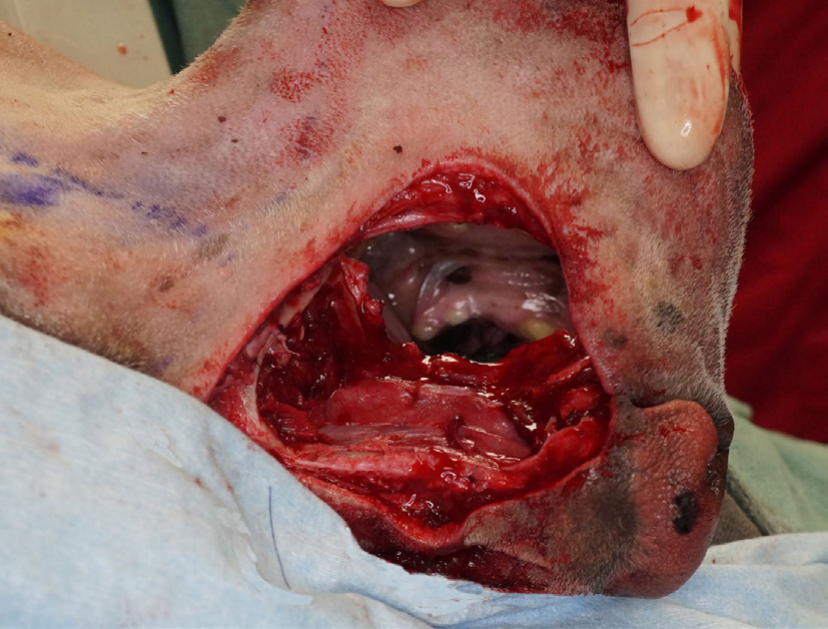
Intraoperative photograph revealing the communication of the intraoral and extraoral approaches after the composite resection has been removed.

**Figure 5:**
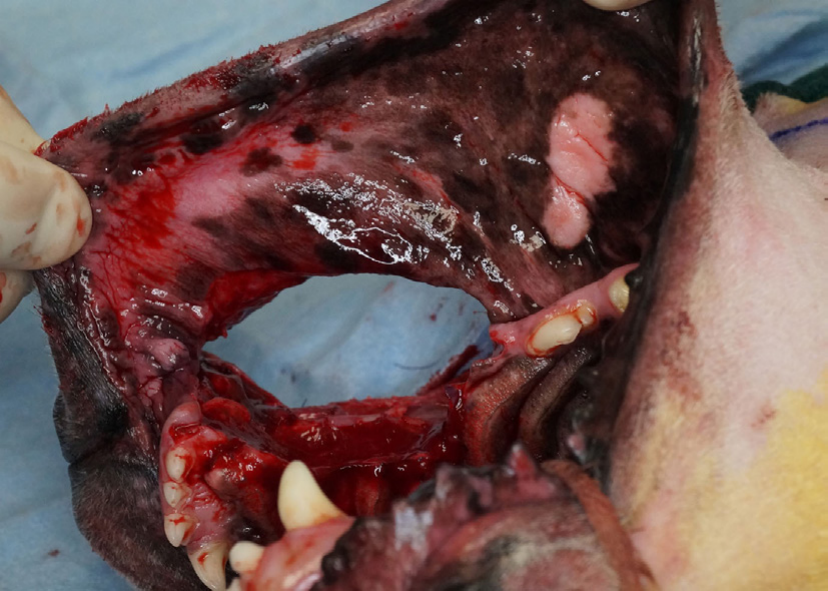
Intraoperative photograph revealing the communication of the intraoral approaches after the composite resection has been removed.

**Figure 6:**
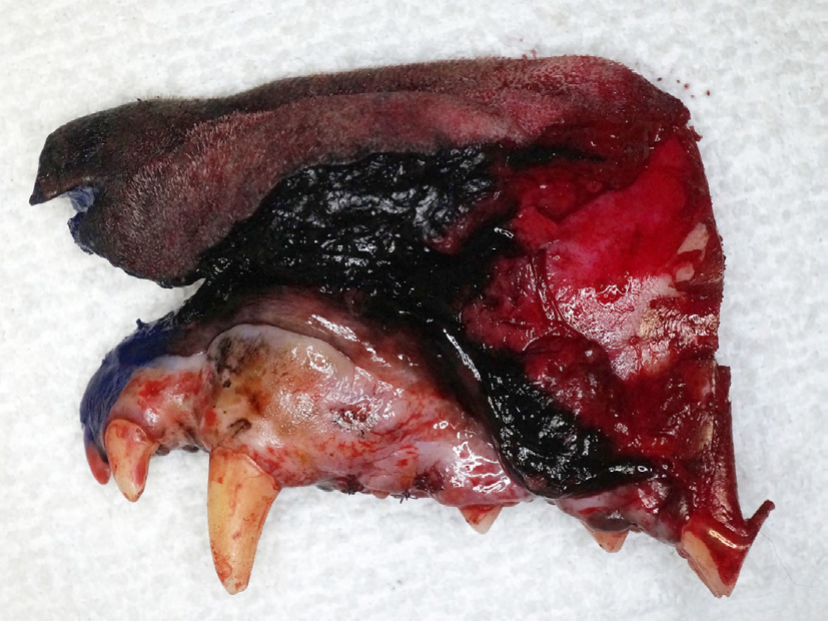
Intraoperative photograph of the composite resection after removal. Note the nasal planum and dermis at the dorsal aspect and the teeth and oral mucosa at the ventral aspect.

**Figure 7:**
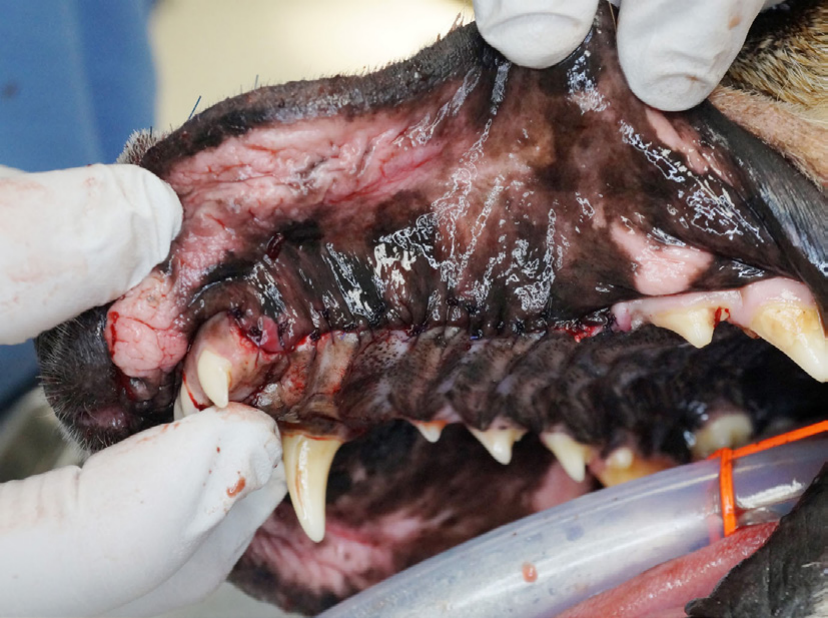
Intraoperative photograph revealing the simple advancement flap of mucosa utilized to close the intraoral approach and recreate the oral vestibule.

**Figure 8:**
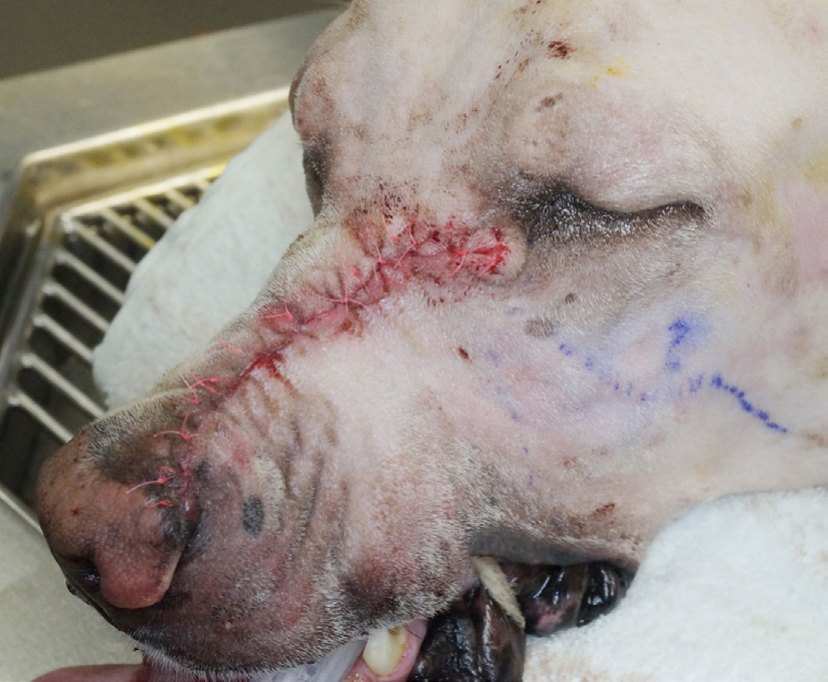
Intraoperative photograph revealing the simple advancement flap of dermis utilized to close the extraoral approach. Note: written informed consent was obtained from the owners for the publication of this image.

The airway was secured by orotracheal intubation immediately following induction of general anesthesia. To minimize oral bacterial load and contamination, the dentition in the region of the surgical approach was scaled and polished, and an oral antiseptic rinse was applied, before surgery. Dogs were positioned in a modified dorsal recumbency position with the head positioned slightly lateral, surgical side up. The entire maxilla was clipped and prepared for aseptic surgery from the medial canthus of the eye to the nasal planum and from the ipsilateral mucocutaneous junction to at least 2 cm (dictated by tumor location) beyond the dorsal midline of the muzzle. A sterile surgical pen was used to outline surgical margins of 2 cm based on the combination of visible tumor size and osseous extent as determined from computed tomographic (CT) studies. The extraoral surgical margins were inked on the skin of the dorsolateral muzzle, and the intraoral margins were marked on the vestibular oral mucosa, gingiva, and palatal mucosa (Figures 1 and 2). The surgical approach began with an extraoral ellipsoid skin incision along the surgical markings with a #10 scalpel blade. Once the skin along the dorsolateral muzzle was incised, a combination of sharp and blunt dissection was used to incise the subcutaneous tissue and the superficial and deep muscles of the lip, namely, the levator nasolabialis and orbicularis oris superficially as well as the levator labii maxillaris and caninus, to approach the underlying maxilla, incisive and nasal bones. A periosteal elevator was used to reflect the periosteum and associated soft tissues off the underlying bones. The ventrolateral component of this ellipsoid skin incision communicated with the dorsolateral extent of the intraoral incision described below (Figure 3). This communication with the intraoral approach created an approximately 5–7 cm wide isthmus of full-thickness upper lip extending caudally from the rostral muzzle to the buccal tissue (Figures 4 and 5). After completion of the extraoral approach, the intraoral approach was commenced and the gingiva, vestibular mucosa and hard palate mucoperiosteum was incised with a #15 scalpel blade along the intraoral surgical markings. A combination of blunt and sharp dissection was used to locate the infraorbital neurovascular bundle as well as the major palatine artery to allow ligation and transection. After fully exposing the underlying bones, a periosteal elevator was used to release the periosteum and finalize the communication between the intraoral and extraoral incisions. A piezoelectric surgical unit was then used intra- and extraorally to create the osteotomies of the hard palate, maxilla nasal and incisive bones. A winged dental elevator was then used to elevate and remove the composite resection en bloc (Figure 6). The remaining isthmus of upper lip was then bluntly dissected to separate the vestibular mucosa from the skin and labionasal muscles (levator nasolabialis, orbicularis oris, levator labii maxillaris, and caninus). A simple advancement flap was utilized to suture the vestibular mucosa to the palatal mucoperiosteum with 4-0 synthetic monofilament absorbable suture material in a simple interrupted pattern (Figure 7). The skin and muscle layers of the upper lip was advanced dorsally and sutured to the dorsal midline of the muzzle with 4-0 synthetic monofilament non-absorbable suture material in a simple interrupted pattern to close the extraoral defect (Figure 8).

## Case Reports

### Case 1

**Figure 9:**
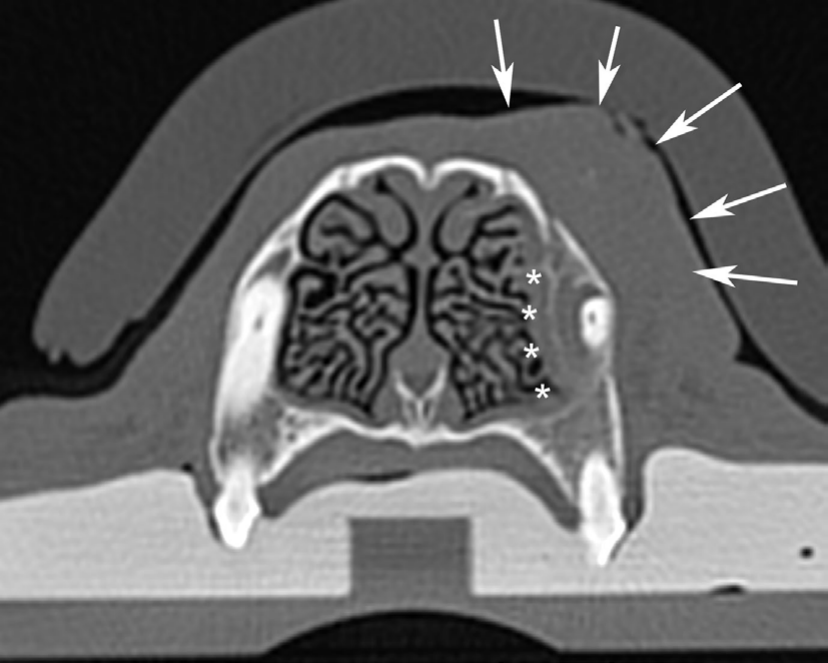
Computed tomographic image of the fibrosarcoma of the rostral maxilla and dorsolateral muzzle in case #1. Note the invasion of the lesion into the medial alveolus of the canine tooth (white asterisks) and the soft tissue mass on the lateral aspect of the maxilla (white arrows). This image is representative of all three cases.

**Figure 10:**
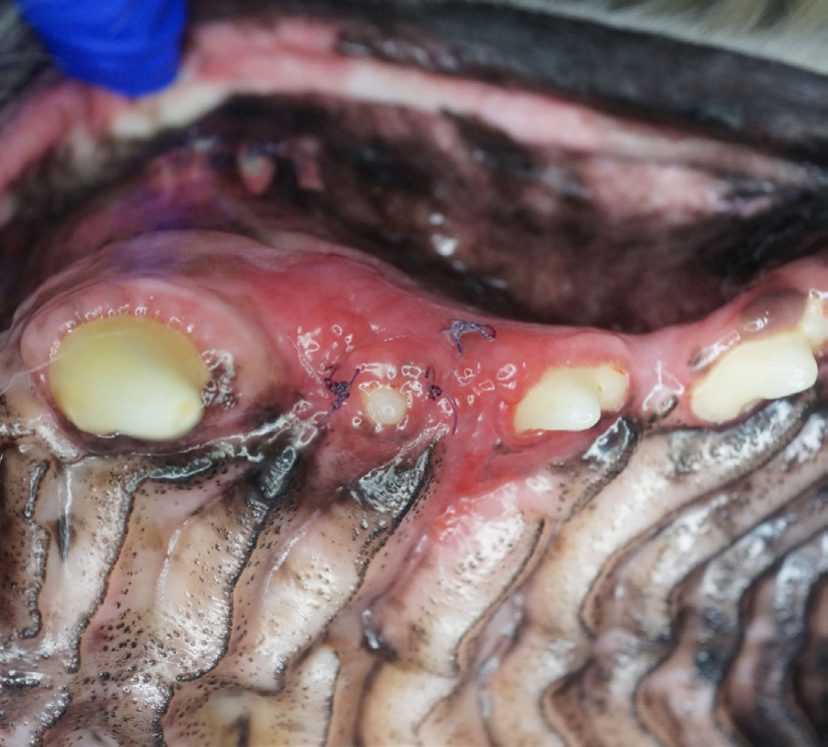
Photograph of the lesion in case #1. Note the clinical appearance of a raised, erythematous, and edematous gingival lesion from the distal canine tooth to the second premolar tooth. This image is representative of all three cases.

An 8-year-old spayed female golden retriever was referred to the medical oncology service for evaluation and of a biopsy-confirmed fibrosarcoma (FSA) of the left rostral maxilla and dorsolateral muzzle. On presentation, the swelling had a rostral to caudal linear diameter of 3.7 cm, and suture material from the referring DVM (rDVM)-acquired biopsy was still present. Absence of metastasis was confirmed *via* lymph node aspiration and thoracic radiography. Head CT revealed a 1.2 cm soft tissue attenuating mass along the left dorsolateral maxilla extending from the mesial aspect of the left maxillary canine tooth to the mesial aspect of the left maxillary second premolar tooth. The dorsal extent of the mass was 0.7 cm from the dorsal midline of the muzzle and ventrally involved the alveolar margin of left maxillary canine tooth. An intraoral lesion was visible from the distal left maxillary canine tooth to the left maxillary second premolar tooth (Figures 9 and 10). Based on the clinicopathologic correlation of the lesions biological behavior, the tumor was determined to be a histologically high-grade, biologically low-grade FSA (H/L FSA).

The case was referred to the dentistry and oral surgery service for surgical treatment. A composite resection utilizing a combined intra- and extraoral approach was performed. The soft tissue resection extended from 1 cm rostral to the medial canthus of the left eye and extended to the nasal planum. From dorsal to ventral, the resection extended from the dorsal midline of the muzzle to approximately 7 cm from the mucocutaneous junction. The osseous resection extended from the mesial aspect of the left maxillary second incisor tooth to the furcation of the left maxillary second premolar tooth. The palatal extent of the resection was the median palatine raphe. Following resection, the distal root of the left maxillary second premolar tooth was extracted, and the wound was closed as described earlier.

Histological evaluation of the resected tissue confirmed the diagnosis of H/L FSA and surgical margins were free of tumor with a narrow margin at the distal aspect of the resection. The dog recovered uneventfully from general anesthesia and was managed overnight in the critical care unit on a continuous rate infusion (CRI) of fentanyl (3 μg/kg/h), ketamine (5 μg/min/h), and lidocaine (25 μg/min/h). A suppository of acetaminophen/codeine (325 mg) and IV ampicillin/sulbactam (900 mg) was also administered. The dog was discharged the following day with a 125 μg fentanyl patch, oral acetaminophen/codeine (325 mg q 8 h), tramadol (5 mg/kg q 8–12 h), and amoxicillin/clavulanic acid (13.75 mg/kg q 12 h). Instructions to feed only canned or softened kibble and to avoid toys or mouth play were given to the owners. At discharge, the dog seemed comfortable and was eating and drinking well.

At the time of oral surgery recheck and skin suture material removal 14 days later, the owners reported the dog was doing very well at home, eating and drinking well, was not experiencing difficulty breathing and experiencing occasional episodes of reverse sneezing. The skin and oral incisions had healed well, and no discharge, redness, swelling, or dehiscence was noted. Skin suture material was removed. At the last recheck of this patient, 18 months postoperatively, there was no evidence of recurrence, and the patient was doing well at home.

### Case 2

A 10-year-old spayed female golden retriever was referred to the medical oncology service for evaluation of a biopsy-confirmed FSA involving the left rostral maxilla and dorsolateral muzzle. Absence of metastasis had been confirmed *via* lymph node aspiration and thoracic radiography by the rDVM before referral. A 3.4 cm, firm swelling was present on the dorsolateral aspect of the left rostral muzzle. Head CT revealed an ill-defined 2.7 cm × 1.8 cm × 2.8 cm soft tissue attenuating mass in the left dorsolateral maxilla extending from the mesial aspect of the left maxillary canine tooth to the mesial aspect of the left maxillary second premolar tooth. The dorsal extent of the mass was 0.6 cm from dorsal midline of muzzle and ventrally involved the alveolar margin of left maxillary canine tooth.

The case was referred to the dentistry and oral surgery service for surgical treatment. A composite resection with a combined intra- and extraoral approach was performed. The soft tissue resection extended from 3 cm rostral to the medial canthus of the left eye to <1.0 cm from the nasal planum. From dorsal to ventral, the resection extended from the dorsal midline of the muzzle to approximately 7 cm from the mucocutaneous junction of the left lip. The osseous resection extended from the mesial aspect of the left maxillary third incisor tooth to the distal root of the left maxillary second premolar tooth. The palatal extent of the resection was approximately 1 cm palatal to the dental arch. Following resection, the distal root of left maxillary second premolar tooth was extracted, and the wound was closed as described earlier.

Histopathological evaluation of the resected tissue confirmed the diagnosis of FSA and tumor-free margins. The dog recovered uneventfully from general anesthesia and was managed overnight in the critical care unit on an intravenous (IV) CRI of fentanyl (3 µg/kg/h), lidocaine (13 µg/min/h), and ketamine (5 µg/min/h). Ampicillin/sulbactam (1,050 mg IV q 12 h) was also administered. The dog was discharged the following day with a 100 mcg fentanyl patch, oral tramadol (4 mg/kg q 8–12 h), and clindamycin (5 mg/kg q 12 h). The owners were instructed to feed only canned or softened kibble and to avoid toys or mouth play. At discharge, the dog seemed comfortable and was eating and drinking well. At the time of oral surgery recheck and skin suture material removal 14 days later, there was no evidence of dehiscence, incisions were healing well, and a good cosmetic appearance was apparent. Continued follow-up was done with rDVM. The patient was euthanized 11 months after surgery for renal failure unrelated to the oromaxillofacial neoplasia. At that time, no evidence of recurrence was present.

### Case 3

A 9-year-old neutered male Labrador retriever was referred to the medical oncology service for evaluation of a mass on the left rostral maxilla and dorsolateral muzzle. On presentation, the swelling had a diameter of 3.3 cm, round and firm. The mass could be appreciated intraoral at the apex if 204 and extraorally ~1.5 cm caudal to the nasal planum. The patient was placed under general anesthesia for head and thoracic CT as well as incisional biopsy and regional lymph node aspiration. Regional lymph node aspiration showed reactive lymphoid tissue and the thoracic CT showed atelectasis, consistent with general anesthesia, with no evidence of pulmonary metastasis or intrathoracic lymphadenopathy. Head CT revealed a 4.6 cm soft tissue attenuating mass along the left dorsolateral maxilla extending from the mesial aspect of the left maxillary canine tooth to the mesial aspect of the left maxillary second premolar tooth. The dorsal extent of the mass was 1.5 cm from the dorsal midline of the muzzle and ventrally involved the alveolar margin of the left maxillary canine tooth. Results of histological examination revealed a histologically low-grade FSA with mild bony infiltration. Based on the clinicopathologic correlation, the tumor was determined to be a histologically low-grade, biologically high-grade FSA (H/L FSA).

The case was referred to the dentistry and oral surgery service for surgical treatment. A composite resection utilizing a combined intra- and extraoral approach was performed. The soft tissue resection extended from 1 cm rostral to the medial canthus of the left eye and extended into the left lateral nasal planum. From dorsal to ventral, the resection extended from the dorsal midline of the muzzle to approximately 3.5 cm from the mucocutaneous junction. The osseous resection extended from the mesial aspect of the left maxillary second incisor tooth to the furcation of the left maxillary fourth premolar tooth. The palatal extent of the resection was the median palatine raphe. Following resection, the distal root of the left maxillary fourth premolar tooth was extracted and the wound was closed as described earlier.

Histological evaluation of the resected tissue confirmed the diagnosis of H/L FSA and surgical margins were free of tumor with a narrow margin at the palatal aspect of the resection. The dog recovered uneventfully from general anesthesia and was managed overnight in the critical care unit on a CRI of sufentanil (0.3 µg/kg/h) and ketamine (5 µg/min/h). A subcutaneous injection of 2.2 mg/kg carprofen (95 mg) and IV ampicillin/sulbactam (950 mg) was also administered. The dog was discharged the following day with a 150 mcg fentanyl patch, oral carprofen (93.75 mg q 12 h), tramadol (5 mg/kg q 8–12 h), and amoxicillin/clavulanic acid (13.75 mg/kg q 12 h). Instructions to feed only canned or softened kibble and to avoid toys or mouth play were given to the owners. At discharge the dog seemed comfortable and was eating and drinking well.

At the time of oral surgery recheck and skin suture material removal 14 days later, the owners reported the dog was doing very well at home, eating and drinking well, was not experiencing difficulty breathing and experiencing occasional episodes of reverse sneezing. The skin and oral incisions had healed well, and no discharge, redness, swelling, or dehiscence was noted. Skin suture material was removed. At the last recheck of this patient, 3 months postoperatively, there was no evidence of recurrence, and the patient was doing well at home.

## Discussion

Composite resections of the oromaxillofacial region may result in considerable cosmetic and functional deformity. Resections that involve the muzzle have traditionally been achieved by extending the extraoral incisions through the mucocutaneous junction of the lip and into the oral cavity, in many cases unnecessarily resecting the upper lip adjacent to the tumor (1). The resultant defect requires reconstruction of the lip with distally based upper lip/buccal advancement flaps or axial pattern flaps (superficial cervical, caudal auricular, superficial temporal, and angularis oris) (2–6). While axial pattern flaps are useful, and certainly have their place, they are not without substantial morbidity and potential complications. Thus, when possible, steps to simplify flap design and avoid potential complications, such as flap tension, ischemic necrosis, and infection at the donor site, are prudent.

The surgical approach and wound closure utilized in the three dogs of this report has been very briefly described in a veterinary surgery textbook in which it was called the “upper labial pull-down technique” (1). However, surgeon experience utilizing the technique in clinical patients has not been documented. Our experience with the technique has been very positive. The approach has several potential advantages. Sparing the mucocutaneous junction-containing upper lip would be expected to minimize tension, minimize the risk of postoperative ischemic necrosis and dehiscence, minimize unnecessary complications associated with large dermal flaps, and provide superior postoperative cosmesis. Perhaps most important, we avoid creating an overly large postoperative wound. As a result, the surgeon may utilize a simple, wide-based advancement flap, which provides many potential benefits over distally based upper lip/buccal advancement flaps or axial pattern flaps. The rostral extent of the postoperative wounds in the patients described here occurred as far rostrally as the nasal planum and one involved a small degree of the nasal planum. Advancing a distally based upper lip/buccal advancement flap this far rostrally would likely exceed the recommended 2:1 flap length to base width ratio resulting in excessive tension, which would significantly increase the risk of ischemic necrosis and dehiscence (7).

Axial pattern flaps have greater potential to reach distant wounds and have a reported overall survival of 89–100% (5, 8–12). However, distal ischemic necrosis has a reported frequency of up to 33% and remains a concern (2). The superficial cervical, caudal auricular, and superficial temporal axial pattern flaps are limited in their ability to reliably reach the nasal planum and may be prone to distal necrosis (1–6). In addition, because of the lack of oral mucosa, axial pattern flaps replace the normal oral mucosa of the oral cavity with hirsute skin, which creates additional complications (13). Of all the known axial pattern flaps for the oromaxillofacial region, the angularis oris axial pattern flap shows the most promise for reaching the nasal planum (2). However, to achieve this length, the flap must be dissected into an anatomically complex region. Thus, potential complications of the angularis oris axial pattern flap include damage to the branches of the facial nerve, branches of the trigeminal nerve, parotid salivary duct, and facial vein (2). While meticulous surgical dissection can help avoid most of these complications, when possible the use of a simple advancement flap, as described here, avoids the chance of these complications altogether.

The remaining lip tissue resulting from the approach described here, provides, in essence, a bipedicle advancement flap supplied by the preserved lateral nasal artery and the inferior labial vein. The lateral nasal artery, a branch of the infraorbital artery, courses rostrally from the infraorbital foramen in the inferior one-third of the lip. Thus, the flap is vascularized by a substantial arterial supply significantly minimizing any risk of ischemic necrosis. However, because mucosa must be separated from the flap to close the intraoral defect, the risk of ischemic necrosis cannot be fully eliminated.

**Figure 11:**
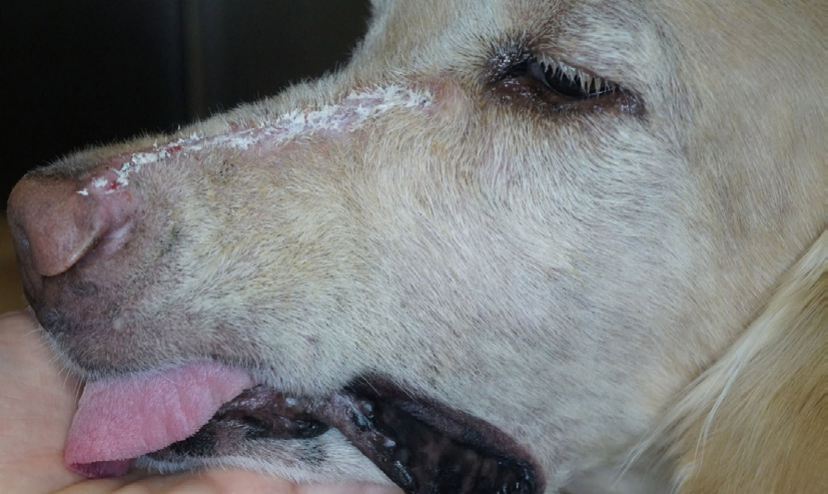
Fourteen-day follow-up photograph depicting the typical cosmetic outcome. Note: written informed consent was obtained from the owners for the publication of this image.

While proper wound healing and acquisition of a quick return to proper function is critical to clinical success, often a client’s willingness to pursue a major oromaxillofacial resection is based significantly on the possibility of achieving good postoperative cosmesis. The described technique provides excellent cosmesis (Figure 11). The technique of sparing an isthmus of normal upper lip tissue allows for primary closure with a simple advancement flap and minimal dissection. As a result, we were able to avoid the use of axial pattern flaps, which often provide less than desirable cosmetic outcomes. These poor cosmetic outcomes include discrepancies in hair coat length, direction, and color (2). In addition, we were able to maintain normal mucocutaneous junction and oral mucosa in the defect and avoid complications associated with introducing hirsute skin into the oral cavity.

In conclusion, the upper lip-sparing technique utilizing a combined extra- and intraoral approach to composite resections of rostral maxilla and dorsolateral muzzle, we detail here provides a more conservative approach that reliably allows primary closure of the resultant wound with a simple advancement flap. As a result of avoiding other local or regional flaps for lip reconstruction, this technique minimizes the typical associated complications and provides an excellent cosmetic outcome.

## Author Contributions

The concept for this manuscript was primarily the responsibility of JS. Both JS and AT contributed approximately equally to the writing.

## Conflict of Interest Statement

The authors declare that the research was conducted in the absence of any commercial or financial relationships that could be construed as a potential conflict of interest.
